# Is Extra Virgin Olive Oil the Critical Ingredient Driving the Health Benefits of a Mediterranean Diet? A Narrative Review

**DOI:** 10.3390/nu15132916

**Published:** 2023-06-27

**Authors:** Mary M. Flynn, Audrey Tierney, Catherine Itsiopoulos

**Affiliations:** 1 Department of Medicine, The Miriam Hospital, Brown University, 164 Summit Ave., Providence, RI 02912, USA; 2 Health Implementation Science and Technology Research Group, Human Nutrition and Dietetics School of Allied Health, Health Research Institute, University of Limerick, Castletroy, V94 T9PX Limerick, Ireland; audrey.tierney@ul.ie; 3 School of Health and Biomedical Sciences, STEM College, RMIT University, Melbourne 3083, Australia; catherine.itsiopoulos@rmit.edu.au

**Keywords:** extra virgin olive oil, bioactive nutrients, polyphenols, Mediterranean diet

## Abstract

Most chronic diseases are preventable with a healthy diet, although there is debate about the optimal dietary approach. Increasingly more countries are focusing on food-based guidelines rather than the traditional nutrient-based approach. Although there is good agreement on plant foods, controversy remains about the types and amounts of fats and oils. This narrative review aims to systematically summarize and evaluate the latest evidence on the protective effects of extra virgin olive oil (EVOO) on disease risk factors. A systematic search of the relevant literature using PubMed, Cochrane Library, and Embase databases was conducted for the years 2000 through December 2022. A narrative synthesis was then undertaken. Of 281 retrieved articles, 34 articles fulfilled our inclusion criteria and were included. Compared with other dietary fats and low-fat diets, EVOO is superior in the management of clinical biomarkers including lowering blood pressure and LDL-c, increasing protective HDL-c, improving glycemic control, and weight management. The protective effects of EVOO are likely due to its polyphenol content rather than the monounsaturated fat content. It is therefore important to promote the regular use of EVOO in the context of healthy dietary patterns such as the Mediterranean diet for maximal health benefit.

## 1. Introduction

Chronic non-communicable diseases (NCDs) are responsible for the deaths of 41 million people each year, equivalent to 74% of all deaths globally [1]. Over 80% of all premature NCD deaths are due to cardiovascular diseases (CVDs), which account for the large majority (~40%), followed by cancers, chronic respiratory diseases, and diabetes. An estimated 90% of deaths from CVD can be prevented with modifiable risk factors such as healthy dietary patterns rich in plant foods such as the Mediterranean diet. The traditional Mediterranean diet is a plant-based diet rich in seasonal fruits and vegetables, legumes, nuts, whole-grain cereals, moderate servings of oily fish, and fermented dairy such as feta cheese and yogurt and very low in red meat with a preference for white meats and eggs that are free range [2]. One of the most important ingredients of the Mediterranean diet that is consistent across all Mediterranean countries is extra virgin olive oil consumed liberally as the main added fat. There is now widespread agreement that the Mediterranean diet is important in the prevention of chronic diseases, and this is reflected in many dietary guidelines for chronic diseases worldwide [3,4,5,6]; however, there is very little focus on differentiating healthy fats such as EVOO from other fats and oils in dietary guidelines.

The current dietary guidelines of United States health agencies [3] and most dietary guidelines across the UK [7], Ireland [8], and Australia [9] do not distinguish between dietary oils for health benefit or potential harm. Seed oils (corn, flaxseed, safflower, soybean, and sunflower) can readily oxidize due to the high polyunsaturated fat content [10,11,12], which has the potential to initiate and promote disease processes. Oils that are mainly monounsaturated or saturated fat are less likely to oxidize [10], but harm–benefit associations with health would depend on the individual oils.

The habitual use of extra virgin olive oil (EVOO) is associated with decreasing the risk of a range of diverse chronic diseases including cardiovascular disease [13], cerebral vascular accidents [14], and type 2 diabetes [15,16]; both the prevention [17] and reversion [16] of the metabolic syndrome; the prevention of decline in cognitive function [18]; and reductions in the risk of breast [19,20,21,22] and colorectal [23,24] cancers. EVOO has also been shown to lower the risk of obesity [25] and weight gain over time [26] and to improve overall mortality [27]. This suggests that EVOO may be unique among the dietary fats in its ability to decrease the risk of multiple chronic diseases, and hence, its place in dietary guidelines should be emphasized.

While vegetable seed oils are produced by chemical extraction of the oil, EVOO is produced by crushing the olive fruit with no use of chemical solvents and only water at ambient temperature during malaxation, which would preserve the phenols naturally present in the oil. Thus, olive oil is essentially the juice of olive fruit. To be classified as “extra virgin”, the oil must meet chemical and sensory standards [28]. However, the phenol content of the olive oil provides the health benefits [29], as opposed to the fatty acid composition of the oil. The individual phenols and the amount of the phenols present in the olive oil are influenced by the olive variety and the growing, harvesting, and processing of the olive [30]. In addition to health benefits, the phenols and other minor flavor compounds in EVOO give the sensory characteristics of the oil [31]. The phenols in EVOO have been found to be bioavailable with absorption rates of more than 50% [32], and plasma levels increase in a dose-dependent manner based on the total phenol content of the EVOO [33]. There is evidence that the phenols in EVOO will bind to low- [34] and high-density lipoprotein cholesterol [35], which would preserve the phenols in the blood. The attachment of the phenol to lipoproteins may prevent the oxidation of these lipoproteins and would also provide the phenol transport to cells where they can exert their function.

To understand how EVOO decreases the risk of chronic diseases, the effect of EVOO on risk factors for the diseases needs to be assessed. Herein, this review aims to compare the effect of diets that include EVOO with other defined diets that do not include extra virgin olive oil on clinically relevant cardiometabolic risk factors assessed for heart disease, metabolic syndrome, and type 2 DM. Specifically, it will examine the effects on blood pressure, low- and high-density lipoprotein cholesterol, fasting blood glucose, and body weight. It will also assess from published studies the minimum daily amount of EVOO and the shortest time needed to realize improvements in the risk factors.

## 2. Methods

This review involved a systematic search with inclusion and exclusion criteria, quality assessment of the studies that were identified, and summary of the study findings [36]. A literature search of PubMed, Cochrane Library, and Embase was conducted for the years 2000 through December 2022 using the search tool EndNote X9^®^. The search included MeSH terms of “olive oil” with the variables of “blood pressure”; “hypertension”; “glucose”; “insulin”; “insulin resistance”; “cholesterol, LDL”; cholesterol, HDL”; “body weight”; and “weight loss”. The articles retrieved were assessed against inclusion and exclusion criteria. Aligned with the PICO method, the inclusion criteria were original research papers published in English, peer-reviewed journals, human studies (population), randomized controlled trials, testing a known amount of EVOO that was part of the prescribed diet, the extra virgin olive oil being consumed daily (intervention), the comparison being with another defined diet that did not contain EVOO (C), and the cardiometabolic effects of the trials containing EVOO as noted in the search terms above (outcome). Each article was inspected to verify the inclusion of the variable of interest. The exclusion criteria were animal or in vitro studies, lack of randomization, solely postprandial measurements, test of olive oil phenols only, olive oil as a supplement pill, olive oil that had components added, not stated as extra virgin olive oil, stated use of refined or pomace olive oil only, abstracts or presentations, or the article indicating a potential change in medication that could impact one of the variables to be studied. Studies that tested “virgin olive oil” were included if a phenol content was provided. The reference lists from the articles found using searches were manually examined for additional eligible articles to include.

One author (MF) extracted information from the included studies—the population studied, details on the intervention (amount of EVOO and duration of study/EVOO exposure), and effect on outcomes (cardiometabolic risk factor variables)—and evaluated it. The articles retrieved were assessed for quality using the Academy of Nutrition and Dietetics Quality Criteria Checklist (ANDQCC) for primary research [37]. The ANDQCC contains four questions regarding the relevance of the research and ten questions relating to the validity of the research. This checklist evaluates the internal and external bias within the study to determine the quality of the studies’ inclusion/exclusion criteria, data collection and analysis, and the generalizability of the results to grade the quality of the study. A meta-analysis was not performed due to the heterogeneity in the included studies and the difference in the reporting of the outcome variables of interest.

## 3. Results

**Figure 1:**
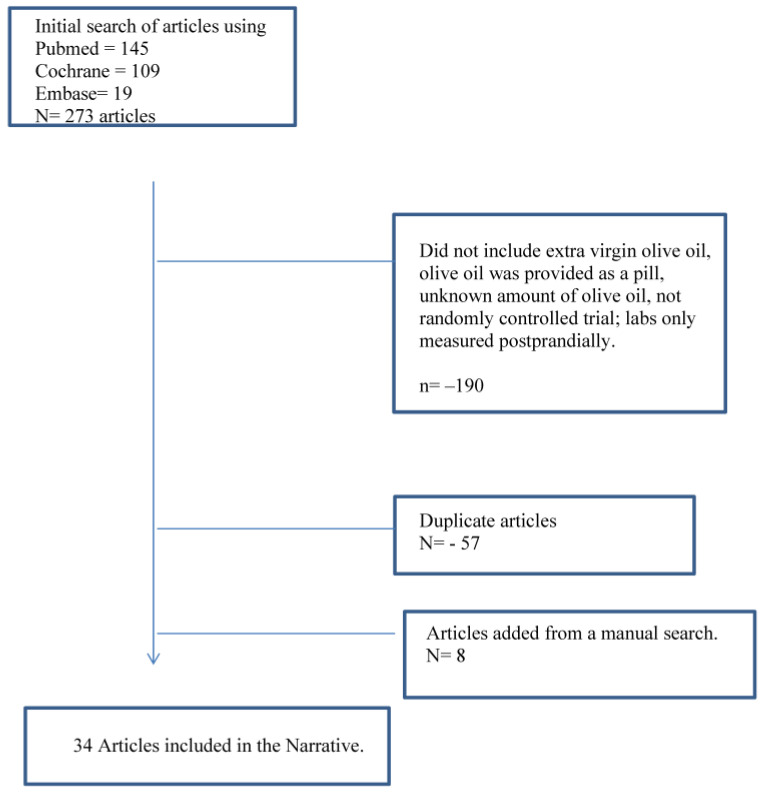
Flow diagram of the literature review processes evaluating studies comparing extra virgin olive oil with diets with other fats, low-fat diets, or olive oils with differing total phenol contents.

Figure 1 summarizes the major elements of this search that met the inclusion criteria. Thirty-four studies are included in this review, and the ANDQCC is presented in Table 1, which shows that all of the studies received a “positive” rating. The results for each risk factor are presented as a comparison of EVOO with another dietary fat, a low-fat (LF) diet, refined olive oil, or EVOO differing in phenol content. The results are presented as they were provided in the journal article. Several of the references were from the Prevencion con Dieta Med (PREDIMED) study, including subgroup studies. The PREDIMED study took place in Spain and enrolled participants from 2003 to 2009. It included 7447 total participants who had either type 2 diabetes or at least three risk factors for cardiovascular disease [38]. Participants were randomly assigned to a low-fat diet (control), a diet with four tablespoons a day of EVOO, or a diet with 30 g a day of nuts. Reports were published at several time points over the course of the trial. This review included papers reporting on the longest time point for a variable and only included an earlier time point if the publication had additional data that were not found in a more recent report.

### 3.1. Blood Pressure

Sixteen RCTs examined the effect of EVOO on blood pressure [39,41,43,44,46,47,48,51,55,60,62,63,65,66,67,70]. Of these, six included the total phenol content of the EVOO studied [39,41,48,62,63,68]. Five stated that participants had hypertension (HTN) [44,47,55,63,65], one had baseline blood pressure that would be classified as hypertensive [48], and three were PREDIMED results that would have included some participants with hypertension [43,46,70]. Table 2provides the information on the RCTs for blood pressure.

For the studies that included participants with HTN, EVOO lowered the systolic blood pressure (SBP) compared with sunflower oil [47,65] but not compared with coconut oil [55]. For the PREDIMED studies comparing the EVOO group with the LF group, DBP was lower in the EVOO group at three months [46], at one year [43], and after four years [70], and SBP was lowered only at three months [46] and one year [43]. An additional study that was not from the PREDIMED trials comparing an LF diet with extra virgin olive oil found no difference in the systolic or diastolic blood pressure between the diets [44]. For the two studies that included both the total phenol content of the olive oil and hypertensive participants, an EVOO with a total phenol of 161 mg/kg [48] and one with 564 mg/kg [63] both lowered SBP, while DBP was lowered only with the 564 mg/kg total phenol olive oil and was borderline significant for the 161 mg/kg total phenols.

For the seven studies that included normotensive participants, three studies reported the olive oil used as “extra virgin olive oil” [51,60,66]. Two reported EVOO lowering only DBP compared with corn [60] or soybean oil [51], and one reported only lowering SBP compared with an intervention that was corn oil, soybean oil, and butter [66]. Three reported a total phenol content of 366 mg/kg, and DBP was lowered compared with refined olive oil [39,41,62]. Of the three studies comparing refined olive oil with 366 mg/kg olive oil [39,41,62], only one of the studies reported a decrease in SBP [39], which was also seen in the Sarapis et al. study that used 360 mg/kg [67], suggesting there may have been different phenols in the oils despite the same total phenol content.

### 3.2. Low-Density Lipoprotein Cholesterol (LDL-c)

Twenty RTCs examined the effect of EVOO on LDL-c [33,40,45,46,47,48,50,55,56,57,58,59,60,61,62,63,64,65,66,67,68]. Of these, nine included the total phenol content of the EVOO studied [33,41,42,50,53,54,61,62,68]. Eight included participants with a mean baseline greater than 120 mg/dL [41,52,55,59,61,62,69,71]. Table 3 displays the RTCs for LDL-c.

For the studies of participants having a baseline LDL-c greater than 120 mg/dL, those listing the oil as “extra virgin olive oil” found EVOO oil decreased LDL-c compared with sunflower oil [59] but not compared with corn oil [71] or coconut oil [55]. Two PREDIMED reports reported baseline LDL-c, one at three months [69] and the other at one year [52]. Comparing those in the EVOO group with those in the low-fat diet group, LDL-c was not different at three months but was lower in the LF group at one year. Despite no difference in LDL-c, the EVOO group had lower apoprotein B-100 at three months [69], while there was no difference at one year [52]. In addition, the LF diet group at one year had more atherogenic smaller LDL-c particles [52]. Two studies that compared refined olive oil with one with a total phenol content of 366 mg/kg reported the higher phenol content olive oil lowering LDL-c [41,62]. While use of an EVOO with a total phenol content of 150 mg/kg did not lower the total LDL-c, it did decrease LDL oxidation [61].

For studies with a baseline LDL-c less than 120 mg/dL, one compared EVOO with flaxseed oil and found lower LDL-c after flaxseed oil [56]. Five studies compared refined olive oil with known total phenol content [33,42,53,54,68], with four reporting no difference in the total LDL-c between the comparative groups [33,42,53,68]. Two of these measured LDL oxidation and found a total phenol content of 366 mg/kg reduced LDL oxidation [42,53]. One study with baseline LDL-c less than 100 mg/dL comparing refined olive with EVOO with 500–700 mg/kg total phenols found the EVOO decreased LDL-c after six weeks [54]. The study by Hernaez et al. [53] also reported a decrease in total LDL particles and apo B100 with a total phenol content of 366 mg/kg compared with a refined olive oil.

### 3.3. High-Density Lipoprotein Cholesterol (HDL-c)

Twenty-one RTCs examined the effect of EVOO on HDL-c [33,35,40,41,42,49,50,51,54,55,56,57,58,59,60,61,62,64,65,68,69]. Of these, eight reported the total phenol content of the EVOO [3,35,41,42,50,54,61,62,68]. Sixteen studies reported a baseline HDL-c, and of these, twelve included men with a mean HDL-c of 45 mg/dL or greater [35,41,42,50,55,56,57,60,61,62,68,69]. One study from Brazil reported baseline HDL-c of less than 25.0 mg/dL, which was the only published study retrieved with the search that included participants with an HDL-c below a healthy range, and HDL-c was not changed after nine weeks compared with soybean oil [51]. Table 4 outlines the RTCs for HDL-c.

Studies that compared the effect on HDL-c of other dietary fats with EVOO report inconsistent results. Flaxseed oil [56], sunflower oil [59], soybean oil [51], and corn oil [60] may all produce similar HDL-c compared with EVOO, while compared with sunflower oil [65], HDL-c levels may decrease. In a study comparing the effect on HDL-c of coconut, butter, and extra virgin olive oil, coconut oil produced the highest HDL-c levels [55], although the mean baseline HDL-c was exceptionally high for all participants (70–77 mg/dL).

Compared with lower-fat diets, three of the five studies showed an increase in HDL-c for EVOO [49,64,69]. Studies testing refined olive oil to known total phenol content suggest specific phenols may be important to assessing change. For example, while two studies comparing refined olive oil with olive oil with a total phenol content of 366 mg/kg found no difference in HDL-c after three weeks [41,62], another study testing a total phenol of 150 mg/kg found higher HDL-c levels compared with refined, also after three weeks of study [61]. Two studies compared HDL-c changes with a refined olive oil and two different levels of total phenols, one less than 200 mg/kg and one greater than 300 mg/kg. Both found that HDL-c increased linearly with the phenol content [33,42]. Interestingly, five studies that compared refined olive oil with olive oil with a total phenol content greater than 300 mg/kg found no difference in the total HDL-c level [35,41,54,62,68], but for two of these studies [35,68] the male participants had a mean baseline HDL-c greater than 50 mg/dL. Despite no change in total HDL-c, a 366 mg/kg total phenol olive oil increased both the level of HDL-2 and the efflux capacity of the HDL-c, suggesting that even if total HDL-c does not increase with EVOO, the inclusion of EVOO may improve HDL function [35].

### 3.4. Fasting Blood Glucose

Fifteen RCTs examined the effect of EVOO on fasting blood glucose (FBG) [41,43,44,46,48,49,50,51,55,56,57,58,59,62,64], and eight included results for insulin [44,46,49,50,51,58,59,64]. Five studies included the phenol content of the EVOO tested [41,44,48,50,62]. Only one RCT testing the effect of EVOO on FBG stated that the participants were type 2 DM [59]. As the PREDIMED trial included people with type 2 diabetes, at least some of the participants in the two RCTs of PREDIMED with results at both three months [46] and one year [43] would have had type 2 diabetes. Table 5 presents the RTCs for FBG.

The only study where all participants had type 2 diabetes reported the oil used as “extra virgin” [59]. Compared with sunflower oil, the EVOO decreased both FBG and insulin in two weeks of study. A study comparing EVOO with soybean oil for participants with normal baseline FBG found no difference in FBG after nine weeks, but insulin was lower in the EVOO group, and HOMA-IR (Homeostatic Model Assessment of Insulin Resistance) was borderline lower [51]. In the PREDIMED trial, FBG, insulin, and HOMA-IR were all lower in the EVOO group compared with the LF diet group at three months [46]. An assessment at one year only reported FBG and showed a larger decrease in FBG for the EVOO group compared with the LF group [43].

Three of the studies that included total phenol content compared with refined olive oil found no difference in FBG for participants with normal baseline FBG for a total phenol content of 161 mg/kg [48] or 366 mg/kg total phenols [41,62]. Two studies compared a known phenol content olive oil with an LF diet, and the one with 172 mg/kg found no difference for FBG or insulin [44], while the other using 625 mg/kg resulted in a decrease in both FBG and insulin [50].

### 3.5. Body Weight

Six RCTs examined the effect of EVOO on weight loss [41,45,49,50,51,62]. Of these, three reported the total phenol content of the EVOO tested [41,50,62]. Four included participants with a mean baseline BMI greater than 25.0 kg/m^2^ [45,49,50,51]. Table 6 presents the RTCs for body weight.

Only one study compared EVOO with another fat source, which was soybean oil for weight loss [51]. While the weight loss was the same between the interventions for nine weeks of weight loss, the participants assigned to EVOO lost more body fat as measured by dual energy X-ray absorptiometry (DXA).

Three studies compared an LF diet to a diet with EVOO [46,49,50]. Two were crossover studies that compared a diet rich in EVOO (three tablespoons a day) with LF diets for eight weeks of weight loss and then six months of follow-up where the participants self-selected one of the diets. In a study of breast cancer survivors [49], of the 15 who started with the EVOO diet, 12 achieved the weight loss goal of at least 5% from baseline, versus four of the 13 who started with the low-fat diet. Despite the greater weight loss, the women reported consuming more total energy while on the EVOO diet (EVOO: 1466 ± 201 kcals vs. LF: 1142 ± 208; *p* < 0.001). Nineteen of the 22 women who continued after the initial weight loss phase selected the olive oil diet for follow-up. The six months of follow-up resulted in an increase in both HDL-c level and blood levels of measured carotenoids compared with the end of the active weight loss of eight weeks. In a study of 18 men with recurrent prostate cancer on hormone therapy, the weight loss was comparable for the LF diet compared with the olive oil diet, but again participants reported consuming more total energy while on the olive oil diet (EVOO: 1916 ± 482 kcal vs. 1442 ± 477; *p* < 0.00) [50]. Thirteen of the 18 participants selected the olive oil diet for follow-up where weight loss was maintained, and there was no further improvement in laboratory measures.

The PREDIMED study assessed weight loss between the diets and found after 4.8 years of study, those assigned to the EVOO diet lost more weight than those assigned to the low-fat diet and had a greater reduction in waist size [45]. In addition, the EVOO participants reported consuming 141 (CI 95% 97–185) more total calories per day compared with the low-fat group (*p* < 0.001).

Two short-term (each three weeks) studies compared weight loss on refined olive oil with an EVOO with 366 mg/kg; both were healthy men with baseline body mass index (BMI) < 25.0 kg/m^2^ [41,62]. One study found slightly more weight loss for the EVOO with a phenol concentration of 366 mg/kg [41]. The other one found no difference in weight change between the oils [62].

### 3.6. Quality Assessment

All articles were given an overall positive rating according to the ANDQCC. The only quality assessment that was not predominately positive was blinded treatment with 16 of the 34 studies not being blinded. This is not an uncommon finding in dietary trials due to the difficulty in blinding food-related interventions. The remaining assessment questions were overwhelmingly positive for all studies.

## 4. Discussion

Compared with other dietary fats or low-fat diets, there is evidence to support EVOO improving SBP in hypertensive patients [43,46,47,65] and also in patients with clinically normal SBP [66]. Studies reporting the total phenol content of the olive oil suggest that specific phenols may be important as compared with a refined olive oil; one with a total phenol of 161 mg/kg improved SBP in patients with HTN [48], while two studies with total phenols greater than 300 mg/kg did not improve SBP in patients with mild HTN [41,67]. Compared with other dietary fats or low-fat diets, EVOO can decrease LDL-c for baseline values greater than 120 mg/dL [59] and increase HDL-c [49,55,64,65] with a linear increase with higher total phenol content [33,42], and diets including daily EVOO are effective for weight loss [41,45,49] and long-term weight management [49,50,72]. In addition, an EVOO with a total phenol content of at least 150 mg/kg has been shown to decrease LDL oxidation [42,53,61]. The effect of EVOO on FBG compared with other diets is not clear, as few studies have included participants with elevated baseline FBG or type 2 diabetes. However, compared with a lower-fat diet, daily EVOO can improve insulin sensitivity as measured by HOMA-IR [46,50,64]. The shortest time to benefit and the minimum daily amount of EVOO required to improve both SBP and DBP [39], LDL-c [41], and HDL-c [42,61] as reported in the literature are three weeks and 25 mL (approximately two tablespoons) a day, with both diastolic blood pressure and LDL-c possibly needing a total phenol content greater than 300 mg/kg to see a benefit. It is possible that FBG could be improved in as few as two weeks with 25 mL of EVOO [59]; however, more studies on type 2 diabetes would be needed to confirm this.

The relationship of EVOO to decreasing the risk of CHD is potentially through the ability of EVOO to improve the clinical biomarkers for CHD of blood pressure, LDL-c, and HDL-c. While a diet that includes vegetable seed oil may decrease LDL more than EVOO [56,60], a diet that includes daily EVOO will produce healthier LDL as the particles will be larger [52] and are less likely to be oxidized [42,61]. In addition, EVOO has been shown to decrease apo protein B-100 [69], indicating fewer LDL particles. Daily use of EVOO increases HDL-c [49,61,64] and will also improve HDL function [35]. In addition to the clinical biomarkers of blood pressure and lipoproteins, EVOO improves other biomarkers that have been recently related to CHD risk. For example, EVOO decreases inflammation as measured by C-reactive protein compared with refined olive oil [63], a low-fat diet, or a diet that includes tree nuts [73]. Compared with a low-fat diet [17,74] or refined olive oil [63], EVOO improves endothelial function and decreases platelet aggregation by improving several factors related to blood clotting [29].

The ability of EVOO to decrease the risk of the metabolic syndrome and type 2 DM is related to its effects on FBG, insulin, and HOMA-IR, which can all be improved by EVOO compared with either a diet that includes an oil rich in polyunsaturated fat (sunflower oil) [59] or a low- fat diet [43,46,50]. One study that compared an EVOO-rich diet with a sunflower oil diet indicated that EVOO improves insulin-stimulated glucose transport in adipocytes [75]; thus, EVOO may improve insulin sensitivity. While more RCTs are needed of participants who have type 2 diabetes or with elevated FBG and to report the total phenol content of the EVOO used to ensure it is EVOO, these results are promising. Based on the benefits of EVOO seen in current studies, studies that compare EVOO with other oils for treatment of type 2 DM would be also useful.

A diet that includes daily EVOO may be an effective alternative for weight loss and weight management, decreasing the risk and progression of chronic diseases. Compared with a lower-fat diet that does not include EVOO, a diet with three [49] or four [45] tablespoons per day of EVOO may produce greater weight loss than a lower-fat diet. In addition, an EVOO-rich diet may be more acceptable for long-term use [49,50]. Even if the weight loss from a diet that includes daily EVOO is equivalent to one that includes vegetable seed oil, EVOO may produce more fat loss [51]. In vitro and animal studies suggest that the potential mechanisms for the additional fat loss are the results of the phenols in EVOO reducing adipocyte proliferation [76] and enhanced thermogenesis from the activation of brown fat tissue [77]. Animal studies suggest beneficial effects of oleanoic acid and other triterpenoids [78], but human studies are lacking. In addition, compared with a lower-fat diet, a diet with daily EVOO may allow for a greater total energy intake for equivalent weight loss [45,49,50].

The current standards for extra virgin olive oil do not require measurement of phenols or tests that would indirectly assess the phenol content. The US FDA currently attributes the health benefits of EVOO to the monounsaturated fat content of the oil [79]. Yet studies with oils labeled “olive oil”, “refined olive oil”, or “pomace”, which are all rich in monounsaturated fat but lacking phenols, show these olive oil classifications do not provide health benefits compared with oils that would qualify as “extra virgin” or have the phenol content listed [80]. This provides strong evidence that the monounsaturated fat is not the source of the health benefits found with EVOO. Of the 35 RTCs currently available that examine the health benefits of EVOO, only 15 include the phenol content of the oil. The interpretation of studies that simply list the olive oil as “extra virgin” may require caution as the phenol content is not known. The phenol content of extra virgin olive oil is highest in olive oil made close to the harvesting of the olive and will decrease with age and storage [81]. Thus, for maximum health benefits the EVOO should be produced and consumed as close to harvesting the fruit as possible. As the phenol content of the olive oil will decrease over time, and producers who have their olive oil tested for phenol content would do so when the olive oil is first made, it is not likely bottles of EVOO will have the phenol content listed on them. However, if the olive oil analysis includes the measurement of pyropheophytins (PPP) and 1,2-diacyl-glycerols (DAG), which are currently included in the standards for both California [82] and Australia [83] for olive oil, these would provide information on the freshness of the olive oil, which would relate to the phenol content [84].

### 4.1. Clinical Implications

In prescribing diets to decrease the risk or treatment of a chronic disease, EVOO would be a far superior choice compared with other dietary fats, low-fat diets, or refined olive oil. The daily use of EVOO starting at approximately two tablespoons a day will improve a plethora of risk factors in as few as three weeks. In addition, recommending the use of EVOO to cook vegetables will increase the absorption of carotenoids [85], which are fat-soluble, and could increase vegetable consumption by increasing their palatability [49,86]. Vegetables in Mediterranean countries are traditionally cooked in extra virgin olive oil, which has been shown to transfer the olive oil phenols to the vegetables, which increases the antioxidant capacity of the meal [87].

### 4.2. Strengths

This review only used RCTs, and all of the 34 articles included have a positive score for quality assessment. The included studies compare extra virgin olive oil with other dietary fats and low-fat diets and by olive oil phenol content, thus proving a broad practical comparison. There are no published reviews that focus specifically on the ability of a specific daily amount of extra virgin olive oil to improve risk factors for chronic diseases. The published work to date typically includes all types of olive oil when assessing health benefits [80,88], and the current study indicates this would not be appropriate, as it is only extra virgin olive oil that would provide health benefits.

### 4.3. Limitations

There are several limitations in our paper relating to the currently available RCTs comparing EVOO with other diets that were included in this review. A major limitation is that most of the studies do not include the phenol content of the olive oil, so studies simply listing “extra virgin olive oil” should be interpreted with caution. Most of the currently available studies were of short duration, and participants had healthy levels of the risk factor being studied. In addition, most of the studies took place in EU populations and in countries where extra virgin olive oil has been part of diet for centuries. Future research should focus on using an EVOO with a known phenol content, participants with unhealthy values for the risk factor(s) being tested, a larger sample size, and populations outside of the EU. As most of the benefits were realized in three weeks or less, longer time periods might not be critical; however, it is not currently known if additional benefits could be realized with a longer time of exposure to EVOO.

## Figures and Tables

**Table 1:** Quality Assessment of Studies Using the Academy of Nutrition and Dietetics Quality Criteria Checklist (ANDQCC) [37].

Author	1	2	3	4	5	6	7	8	9	10	Quality Rating
Bondia-Pons et al. [39]	+	+	+	+	−	+	+	+	+	+	Positive
Campos et al. [40]	+	+	+	+	+	+	+	+	+	+	Positive
Castaner et al. [41]	+	+	+	+	−	+	+	+	+	+	Positive
Covas et al. [42]	+	+	+	+	+	+	+	+	+	+	Positive
Domenech et al. [43]	+	+	+	+	−	+	+	+	+	+	Positive
Dos Santos et al. [44]	+	+	+	+	+	+	+	+	+	+	Positive
Estruch et al. (2019) [45]	+	+	+	+	+	+	+	+	+	+	Positive
Estruch et al. (2006) [46]	+	+	+	+	+	+	+	+	+	+	Positive
Ferrara et al. [47]	+	+	+	+	+	+	+	+	−	+	Positive
Fito et al. [48]	+	+	+	+	+	+	+	+	+	+	Positive
Flynn et al. (2010) [49]	+	+	+	+	−	+	+	+	+	+	Positive
Flynn et al. (2017) [50]	+	+	+	+	−	+	+	+	+	+	Positive
Galvao Candido et al. [51]	+	+	+	+	+	+	+	+	+	+	Positive
Hernaez et al. (2017) [52]	+	+	+	+	−	+	+	+	+	+	Positive
Hernaez et al. (2014) [35]	+	+	+	+	−	+	+	+	+	+	Positive
Hernaez et al. (2015) [53]	+	+	+	+	−	+	+	+	+	+	Positive
Khandouzi et al. [54]	+	+	+	+	−	+	+	+	+	+	Positive
Khaw et al. [55]	+	+	+	+	−	+	+	+	+	+	Positive
Kontogianni et al. [56]	+	+	+	+	+	+	+	+	+	+	Positive
Kozic et al. [57]	+	+	+	+	+	+	+	+	+	+	Positive
Kruse et al. [58]	+	+	+	+	−	+	+	+	+	+	Positive
Madigan et al. [59]	+	+	+	+	−	+	+	+	−	+	Positive
Maki et al. [60]	+	+	+	+	+	+	+	+	+	−	Positive
Marrugat et al. [61]	+	+	+	+	+	+	+	+	+	+	Positive
Martin-Pelaez et al. [62]	+	+	+	+	+	+	+	+	+	+	Positive
Moreno-Luna et al. [63]	+	+	+	+	+	+	+	+	+	+	Positive
Paniagua et al. [64]	+	+	+	+	−	+	+	+	+	+	Positive
Perona et al. [65]	+	+	+	+	−	+	+	+	−	+	Positive
Rozati et al. [66]	+	+	+	+	+	+	+	+	+	+	Positive
Sarapis et al. (2020) [67]	+	+	+	+	+	+	+	+	+	+	Positive
Sarapis et al. (2022) [68]	+	+	+	+	+	+	+	+	+	+	Positive
Sola et al. [69]	+	+	+	+	−	+	+	+	+	+	Positive
Toledo et al. [70]	+	+	+	+	−	+	+	+	+	+	Positive
Weinbrenner et al. [33]	+	+	+	+	+	+	+	+	+	+	Positive

+ Answer to validity question was yes. − Answer to a validity question was no.

**Table 2:** The effect of extra virgin olive oil on blood pressure compared with diets with other fats, low-fat diets, and olive oil varying by phenol content.

First Author, Journal, Year, Country	Population	Intervention/Design	Outcomes
**Other Dietary Fats**			
Ferrara, L.A. Arch Inter Med 2000 [47] Italy	n = 23; m/f ^a^ hypertensive 25–70 yrs. BMI ^b^ 26.2 ± 2 kg/m^2^ BP ^c^ < 165/104 mmHg	EVOO ^d^ vs. sunflower oil (SO) 40 g/day m; 30 g/day Crossover 24 wks.	SBP ^e^/DBP ^f^ post-intervention: SBP: EVOO127 + 14 mmHg vs. SO 135 + 13 mmHg; *p* = 0.05 DBP: EVOO 84 + 8 mmHg vs. SO 90 + 8 mmHg; *p* = 0.01 8 on EVOO ceased BP medications
Perona, J.S. Clin Nutr 2004 [65] Spain	N = 62; m/f 31 nl BP; 31 HTN ^g^ 84 ± 7.4 yrs. BMI 28.8 ± 5.2 kg/m^2^	VOO ^h^ (232 mg/kg) vs. sunflower (SO) 60 g/day Crossover 4 wks.	SBP/DBP post-intervention: SBP: HTN: EVOO: 136 ± 10 mmHg vs. SO 150 ± 8 mmHG; *p* < 0.01 nl BP SBP: NS difference (values NA) DBP: NS difference HTN or nl (values NA)
Rozati, M. Nutr Metab 2015 [66] USA	N= 41; m/f healthy 72 ± 1 yrs. BMI 29.1 ± 1 BP: control: SBP 126 ± 2 mmHg; DBP 76 ± 2 mmHg EVOO: SBP 128 ± 3 mmHg DBP 76 ± 2 mmHg	EVOO or combo corn oil (CO), soybean oils (SB), butter (control) 40 g/day Single blind 12 wks.	SBP/DBP—comparing change from baseline values: SBP: EVOO base 128 + 3.7 mmHg to FU 122 + 2 mmHg vs. control base 126.2 ± 2 mmHg to FU 126.2 ± 2 mmHg; *p* = 0.04 DBP: EVOO base 76 ± 2 mmHg to FU 73 ± 1 mmHg vs. Control base 76 + 2 mmHg to FU 73 ± 2 mmHg; *p* = 0.99
Maki, K.C. J Clin Lipidol 2015 [60] USA	N = 54 m/f Healthy 53.8 + 1.3 yrs. BMI: 28.2 + 0.5 kg/m^2^ SBP: 119.5 + 1.6 mmHg DBP: 75.3 + 2.0 mmHg	EVOO or corn oil (CO) 4 tbs/day (35% total fat) Crossover Double blind 21 days	SBP/DBP post-intervention: SBP: EVOO −1.9 + 1 mmHg vs. CO −1.2 + 1 mmHg; *p* = 0.44 DBP: EVOO −1.5 + 0.8 mmHg vs. CO +0.1 + 0.8 mmHg; *p* = 0.04
Galvao Candidio, F.G. Eur J Nutr 2018 [51] Brazil	N = 41; f normotensive EVOO: 26.8 ± 5.0 yrs BMI 30.5 ± 0.60 kg/m^2^ SBP: 115 ± 2.4 mmHg DBP: 74.5 ± 1.9 mmHg Control: 27.2 ± 6.1 yrs BMI 29.7 ± 0.6 kg/m^2^ SBP: 109 ± 2.1 mmHg DBP: 67.5 ± 1.5 mmHg	EVOO vs. soybean (control) 25 mL/day Double blind 9 wks.	SBP/DBP post-intervention: SBP: EVOO −3.9 ± 1.9 mmHg vs. control −3.6 ± 1.5 mmHg; *p* = 0.918 DBP: EVOO −5.1 + 1.6 mmHg vs. control 0.3 + 1.2 mmHg; *p* = 0.01
Khaw, K.T. BMJ Open 2018 [55] UK	N = 91 m/f Healthy EVOO (n = 32): 59.1 ± 6.4 yrs. BMI: 25.0 ± 4.5 kg/m^2^ SBP: 133.1 ± 16.5 mmHg DBP: 78.1 ± 6.7 mmHg Coconut oil (EVco) (n = 29): 59.1 ± 6.1 yrs. BMI: 25.5 ± 4.5 kg/m^2^ SBP: 131.4 ± 18.8 mmHg DBP 79.8 ± 9.3 mmHg Butter (n = 33) 61.5 ± 5.8 yrs. BMI 24.8 ± 3.5 kg/m^2^ SBP: 136.5 ± 18.8 mmHg DBP: 81.0 ± 12.0 mmHg	EVOO vs. coconut oil vs. butter 50 g/day 4 wks.	Mean change from baseline by group: SBP: EVOO −3.7 ± 8.2 mmHg vs. scoconut oil 0.18 ± 11.5 mmHg vs. butter −3.8 ± 11.1 mmHg; *p* = 0.29 DBP: EVOO −0.45 ± 8.5 mmHg vs. coconut oil −2.0 ± 5.7 mmHg vs. butter −1.3 ± 6.2; *p* = 0.81
**Low-fat diet**			
Estruch, R. Ann Intern Med 2006 [46] Spain	PREDIMED N = 722; m/f Type 2 DM or ≥3 CHD risk factors EVOO (n = 257) 68.6 ± 6.9 yrs BMI: 29.7 ± 4.1 kg/m^2^ BP: NA LF ^i^ (n = 257) 69.5 ± 6.1 yrs BMI: 30.2 ± 4.3 kg/m^2^ BP: NA	EVOO vs. nuts vs. (control) LF EVOO = 50 mL/day 3 months	Change in EVOO vs. change LF: SBP: −5.9 mmHg; *p* < 0.001. DBP −1.6 mmHg; *p* = 0.048 Changes greater for those with HTN
Toledo, E. BMC Med 2013 [70] Spain	PREDIMED N = 7447 m/f Type 2 DM or >3 CHD risk factors EVOO (n = 2441) 66.9 + 6.2 yrs. BMI 29.9 + 3.7 kg/m^2^ SBP 148 + 19 mmHg DBP 83 + 10 mmHg LF (n = 2350) 67.3 + 6.3 yrs BMI 30.2 + 4.0 kg/m^2^ SBP 149 + 19 mmHg DBP 82 + 10 mmHg	EVOO vs. nuts vs. (control) LF EVOO = 4 tbs/day 4 yrs.	EVOO vs. LF: SBP: +0.42 mmHg; *p* = 0.35 DBP: −1.41 mmHg; *p* < 0.001
Domenech, M. Hypertension 2014 [43] Spain	PREDIMED N = 235 m/f Type 2 DM or ≥3 CHD risk factors EVOO (n = 78) 66.2 ± 5.8 yrs; BMI: 29.5 ± 3.9 kg/m^2^ SBP 146.2 ± 21.1 mmHg DBP 80.4 ± 10.7 mmHg LF (n = 75) 66.2 ± 6.2 yrs BMI 30.4 ± 3.5 kg/m^2^ SBP 143.8 ± 18.8 mmHg DBP 83.0 ± 9.5 mmHg	EVOO vs. nuts vs. LF 1 year	EVOO vs. LF: SBP: EVOO = −2.3 mmHg; nuts = −2.6 mmHg; LF = +1.7 mmHg; *p* < 0.001 DBP: EVOO = −1.2 mmHg; nuts = −1.2 mmHg; LF = +0.7 mmHg; *p* = 0.017
Dos Santos, J.L. Eur J Clin Nutr 2022 [44] Brazil	N = 204 40–80 yrs. Control = 67 Nuts = 68 Olive oil = 69 Stable CAD Mild HTN; NS between groups, baseline	Control diet = 25% fat Nuts, pecans = 30 g/day Olive oil, total phenol = 172 mg/kg, 30 mL/day 12 weeks of study	Comparison between the groups: NS difference
**Total phenol**			
Fito, M. Atherosclerosis 2005 [48] Spain	n = 40; m Stable CHD Refined → EVOO 69.8 ± 8.4 yrs BMI 28.0 ± 3.0 kg/m^2^ SBP 136 ± 10.9 mmHg DBP 78 ± 8.2 mmHg EVOO → refined 66.0 ± 8.9 yrs BMI 27.0 ± 3/1 kg/m^2^ SBP 136 ± 12.6 mmHg DBP 78.5 ± 12.0 mmHg	14.7 (refined) vs. 161 mg/kg 50 mL/day Crossover 3 wks.	Comparison by phenol content: SBP: Refined: 135.2 ± 6.6 mmHg vs. EVOO 132.6 ± 5.6 mmHg; *p* = 0.001 DBP: Refined: 78.4 ± 6.0 mmHg vs. EVOO 79.6 ± 5.2 mmHg; *p* = 0.06
Bondia-Pons, I. J Nutr 2007 [39] Spain	N = 160; m 5 EU ^j^ cities; North, Central, South (Mediterranean) healthy 33.3 + 1.1 yrs. BMI 23.8 + 2.5 kg/m^2^ SBP North 126.7 ± 2.6 mmHg Central 124.2 ± 2.4 mmHg South 122.0 ± 2.4 mmHg DBP North 80.6 ± 3.3 mmHg Central 78.6 ± 3.2 mmHg South 74.0 ± 3.1 mmHg	2.7 vs. 164 vs. 366 mg/kg phenols. 25 mL/d Crossover Single blind 3 wks.	Baseline to post-intervention by location: SBP: North: base 126.7 ± 2.6 mmHg vs. FU 122.5 ± 2.4 mmHg; *p* < 0.05 Central: base 124.2 ± 2.4 mmHg vs. FU 119.8 ± 2.5 mmHg; *p* < 0.05 South: base 122.0 ± 2.4 mmHg vs. FU 119.6 ± 2.3 mmHg; NS DBP North: base 80.6 ± 3.3 mmHg vs. FU 78.4 ± 3.1 mmHg; *p* < 0.05 Central: base 78.6 ± 3.2 mmHg vs. 75.7 ± 3 mmHg; *p* < 0.05 South: base 74.0 ± 3.1 mmHg vs. FU 72.6 ± 2.9 mmHg; NS
Castaner, O. Am J Clin Nutr 2012 [41] Spain	n = 18; m Healthy 38.2 + 11.5 yrs. BMI 24.7 + 2.9 kg/m^2^ SBP 129 ± 14 mmHg DBP 47 ± 0 mmHg	2.7 vs. 366 mg/kg 25 mL/day Crossover 3 wks.	SBP/DBP change by phenol content: SBP: 2.7 mg/kg 0.88 ± 1.9 mmHg vs. 366 mg/kg −1.6 ± 2.3 mmHg; *p* = 0.361 DBP: 2.7 mg/kg; +2.78 ± 1.7 mmHg vs. 366 mg/kg −1.22 ± 1.04 mmHg; *p* = 0.043
Moreno-Luna, R. Am J Hypertens 2012 [63] Spain	n = 24; f high nl BP or stage 1 HTN 26 yrs (24 to 27 yo) BMI 25.4 kg/m^2^ (23.5 to 27.0 kg/m^2^) SBP 134.4 ± 9.3 mmHg DBP84.6 ± 8.5 mmHg	Refined vs. 564 mg/kg. 60 mL/day Crossover 8 wks.	SBP/DBP change by phenol content: SBP: refined −1.6 ± 8.2 mmHg vs. 564 mg/kg −7.9 ± 9.5 mmHg; *p* < 0.001 DBP refined −2.2 ± 7.2 mmHg vs. 564 mg/kg −6.6 ± 6.6 mmHg; *p* < 0.001
Martin-Pelaez, S. Eur J Nutr 2017 [62] Spain	N = 18; m Healthy 36 ± 11.1 yrs BMI 24.3 ± 3.2 kg/m^2^ SBP 127 ± 14 mmHg DBP 78 ± 9 mmHg	2.7 vs. 366 mg/kg 25 mL/day 65% oleuropein Crossover Double blind 3 wks.	SBP/DBP change by phenol content: SBP 2.7 mg/kg 0.44 ± 1.81 mmHg vs. 366 mg/kg −4.22 ± 1.81 mmHg; *p* = 0.06 DBP 2.7 mg/kg 2.94 ± 1.34 mmHg vs. 366 mg/kg −2.1 ± 1.3; *p* = 0.007
Sarapis, K. Nutrients 2020 [67] Australia	N = 50; m = 17 Healthy 38.5 ± 13.9 yrs BMI 24.7 ± 3.5 kg/m^2^ SBP 120.0 ± 13.4 mmHg DBP 69.9 ± 8.4 mmHg	86 mg/kg vs. 360 mg/kg 60 mL/day Crossover Double blind 3 wks.	Changes from baseline by phenol content: SBP: 360 mg/kg: −2.5 mmHg vs. baseline; *p* < 0.05 86 mg/kg: NS (values NA) DBP: NS difference either phenol amt. (values NA)

^a^ m/f = male/female. ^b^ BMI = body mass index. ^c^ BP = blood pressure. ^d^ EVOO = extra virgin olive oil. ^e^ SBP = systolic blood pressure. ^f^ DBP = diastolic blood pressure. ^g^ HTN = hypertension. ^h^ VOO = virgin olive oil. ^i^ LF = low-fat diet. ^j^ EU = Europe.

**Table 3:** The effect of extra virgin olive oil on low-density lipoprotein cholesterol (LDL-c) compared with diets with other fats, low-fat diets, and olive oil varying by phenol content.

First Author, Journal, Year, Country	Population	Intervention/Design	Outcomes
**Other dietary fats**			
Madigan, C. Diabetes Care 2000 [59] Ireland	n = 11; m ^a^ Type 2 DM 56.0 + 2.5 yrs A1c: 5.7 + 0.86% BMI ^b^ 27.7 + 2.6 kg/m^2^ LDL-c: 123.6 ± 19 mg/dL	EVOO ^c^ vs. sunflower oil (SO) 30 mL/d Crossover 2 wks.	LDL-c comparison by intervention: EVOO: 116.0 + 19.3 mg/dL vs. SO: 123.7 + 19.3 mg/dL; *p* < 0.001
Perona, J.S. Clin Nutr 2004 [65] Spain	N = 62; m/f ^d^ 31 nl BP ^e^; 31 HTN ^f^; 84 ± 7.4 y BMI ^g^ 28.8 ± 5.2 kg/m^2^ LDL-c: NA	VOO ^h^ (232 mg/kg) vs. sunflower (SO) 60 g/day Crossover 4 wks.	LDL-c comparison by intervention: nl BP: VOO 99.2 ± 32.4 mg/dL vs. SO 113.0 ± 35.5 mg/dL; *p* < 0.01 HTN: VOO 105.7 ± 29.3 vs. SO 112.0 ± 27.9; *p* > 0.01
Kontogianni, M.D. Metab Clin Exp 2013 [56] Greece	N = 37 (m = 8) Healthy 25.6 ± 5.9 years BMI 21.9 ± 2.5 kg/m^2^ LDL-c: EVOO: 100 ± 23.2 mg/dL Flaxseed: 103.9 ± 27.0 mg/dL	EVOO vs. flaxseed oil 15 mL Crossover Single blind 6 wks.	LDL-c comparison by intervention: EVOO: baseline 100.0 + 23.2 mg/dL vs. FU ^i^ 100.0 + 23.2 mg/dL Flaxseed: 103.9 + 27.0 mg/dL vs. FU 96.9 + 23.2 * mg/dL *p* = 0.89 for comparisons between diet * *p* = < 0.01 for comparison of baseline LDL-c with FU
Maki, J.C. J Clin Lipidol 2015 [60] USA	N = 54 m/f healthy 53.8 ± 1.3 yrs. BMI: 28.2 ± 0.5 kg/m^2^ LDL-c: 153.3 ± 3.5 mg/dL	EVOO or corn oil (CO) 4 tbs/day Crossover Double blind 21 days	LDL-c comparison by intervention: EVOO −3.5 ± 1.5 mg/dL vs. CO −10.9 ± 1.5 mg/dL; *p* < 0.001
Kruse, M. Mol Nutr Food Res 2015 [58] Germany	N = 19; m Healthy EVOO 58.0 ± 2.6 yrs BMI 29.2 ± 0.7 kg/m^2^ LDL-c: 128.0 ± 11.6 mg/dL Rapeseed/canola 52.0 ± 2.3 yrs BMI: 29.7 ± 0.87 kg/m^2^ LDL-c 130.7 ± 8.9 mg/dL	EVOO or rapeseed (canola) oil (CO) 50 g/day 4 wks.	LDL-c change from baseline: EVOO −5.0 ± 6.6 vs. CO −17.4 ± 4.2; *p* = 0.132
Khaw, K.T. BMJ Open 2018 [55] UK	N = 91; m/f Healthy Coconut oil (n = 28) 59.1 ± 6.1 yrs BMI: 25.5 ± 4.5 kg/m^2^ LDL-c: 135.1 ± 34.7 mg/dL Butter (n = 33) 61.5 ± 5.8 yrs BMI: 24.8 ± 3.5 yrs LDL-c: 135.1 ± 34.7 mg/dL EVOO (n = 30) 59.1 ± 6.4 yrs BMI 25.0 ± 4.5 yrs. LDL-c: 142.8 ± 38.6 mg/dL	Coconut oil vs. butter vs. EVOO 50 g/day 4 wks.	LDL change from baseline: Coconut oil −3.5 ± 18.9 mg/dL vs. EVOO −2.3 ± 15 mg/dL; *p* = 0.74
Galvao Candido, F. Eur J Nutr 2018 [51] Brazil	N = 41; f Control: 27.2 + 6.1 yrs BMI: 29.7 + 0.6 kg/m^2^ LDL-c: 43.6 ± 2.7 mg/dL EVOO: 26.8 ± 5.0 yrs BMI: 30.5 ± 0.6 kg/m^2^ LDL-c: 45.4 ± 3.6 mg/dL	EVOO vs. soybean oil (control) 25 mL/day Double-blind 9 wks.	Change in LDL (mean, SE): EVOO: −0.72 ± 1.44 mg/dL vs. control: −1.0 ± 1.0 mg/dL; *p* = 0.83
**Low-fat diet**			
Flynn, M.M. J Women’s Health 2010 [49] USA	n = 28; f Breast cancer survivors 59.2 ± 6.1 yrs BMI 27.9 ± 2.8 LDL-c: NA	EVOO 3 tbs./day vs. <30% fat (NCI ^j^ diet) Crossover 8 wks.	LDL-c comparison by intervention: EVOO 103 ± 22 mg/dL vs. NCI ^j^ 108 ± 18 mg/dL; *p* = 0.82
Sola, R. Atherosclerosis 2011 [69] Spain	PREDIMED N = 551; m/f Type 2 DM or >3 CHD ^l^ risk factors EVOO (n = 181) 69.3 ± 6.2 yrs. BMI 29.8 ± 4.3 kg/m^2^ LDL-c: 146.2 ± 35.6 mg/dL LF (n = 177) 69.7 ± 6.3 yrs. BMI 30.1 ± 4.3 kg/m^2^ LDL-c: 142.6 ± 33.9 mg/dL	EVOO 50 ml/day Nuts 30 gr/day LF ^k^ 3 months	LDL-c change in EVOO intervention to LF: EVOO vs. LF: −3.2 mg/dL (95% CI ^m^ l −8.4 to 2.0); *p* = 0.23 Apo B: −2.9 (−5.6 to −0.08); *p* = 0.44
Flynn, M.M. J Cancer Therapy 2017 [50] USA	N = 18; m Prostate cancer on surveillance 66.6 ± 5.9 yrs BMI 30.9 ± 2.7 kg/m^2^ LDL-c: NA	EVOO (625 mg/kg) 3 tbs./day vs. Prostate Cancer Foundation diet (PCF) Crossover 8 wks	LDL-c comparison between intervention: EVOO 96.5 ± 24.7 mg/dL vs. PCF 93.8 ± 30.1 mg/dL; *p* = 0.29
Hernaez, A. Mol Nutr Food Res 2017 [52] Spain	PREDIMED N = 210; m/f Type 2 DM or ≥3 CHD risk factors EVOO (n = 71) 66.5 ± 6.34 yrs BMI 30.2 ± 3.96 kg/m^2^ LDL-c: 129 ± 30 mg/dL LF (n = 68) 64.7 ± 6.58 yrs BMI 29.7 ± 3.98 kg/m^2^ LDL-c: 135.0 ± 33.0 mg/dL	EVOO 50 mL/day Nuts 30 gr/day LF 1 year	LDL-c change in LF intervention vs. EVOO: LF −10.5 mg/dL vs. EVOO; *p* = 0.003 LF: no change in apo B; smaller LDL EVOO: increase in size of LDL vs. LF (*p* = 0.021)
Campos, V.P. J Hum Nutr Diet 2020 [40] Brazil	N = 204 Control = 67 (49% m) Nuts = 68 (55% m) Olive oil = 69 (51% m) Stable CAD ^n^ LDL < 100 mg/dL NS between groups at baseline	Control diet = 25% fat Nuts, pecan = 30 g/day Olive oil, total phenol = 172 mg/kg, 30 mL/day 12 wks.	Comparison between groups: NS difference for change in LDL-c
**Total phenol**			
Marrugat, J. Eur J Nutr 2004 [61] Spain	n = 30; m Healthy 54.8 ± 21.4 to 61.0 ± 19.2 yrs BMI: <25.0 kg/m^2^ LDL-c (by diet order) VCR: 135.1 ± 34.7 mg/dL CRV: 142.8 ± 38.6 mg/dL RVC: 150.6 ± 38.6 mg/dL	Refined (R) vs. 68 mg/kg (C = common) vs. 150 mg/kg (V = virgin) 25 mL/day Crossover Double blind 3 wks.	LDL-c change from baseline by phenol content: Refined baseline 131.4 ± 30.9 vs. FU 138.9 ± 34.7 mg/dL; NS 68 mg/kg baseline 138.9 ± 30.9 vs. FU 131.3 ± 30.9 mg/dL; NS 150 mg/kg baseline 135.1 ± 34.7 vs. FU 131.3 ± 2.7 mg/dL; NS 150 mg/kg decreased LDL ox
Weinbrenner, T. J Nutr 2004 [33] Spain	n = 12; m Healthy 21.1 yrs; (20–22 yrs) BMI 22.9 ± 1.7 kg/m^2^ LDL baseline each oil: 10 mg/kg: 77.9 ± 7.7 mg/dL 133 mg/kg: 76.4 ± 7.7 mg/dL 486 mg/kg: 80.1 ± 9.3 mg/dL	10 vs. 133 vs. 486 mg/kg 25 mL/day Crossover Double-blind 4 days	LDL change from baseline by phenol content: 10 mg/kg: 77.9 ± 7.7 → 77.6 ± 7.7 mg/dL 133 mg/kg: 76.4 ± 7.7 → 74.9 ± 7.3 mg/dL 486 mg/kg: 80.3 ± 9.3 → 78.4 ± 7.3 mg/dL *p*> 0.05 for all comparisons
Covas, M.I. Ann Inter Med 2006 [42] Spain	n=200; m Healthy BMI < 25.0 kg/m^2^ 23.7–24.0 mg/m^2^ LDL-c (by diet sequence) 1: 118 ± 35 mg/dL 2: 120 ± 36 mg/dL 3: 113 ± 38 mg/dL	2.7 vs. 164 vs. 336 mg/kg 25 mL/day Crossover 3 wks.	LDL-c change (mean, 95% CI) from baseline by phenol content: 2.7 mg/kg: 0.61 mg/dL (−2.3 to 3.4 mg/dL) 164 mg/kg −0.75 mg/dL (−3.8 to 1.9 mg/dL) 336 mg/kg (−3.1 to 2.3 mg/dL) *p* = 0.74 336 mg/kg: decrease in LDL-ox
Castaner, O. Am J Clin Nutr 2012 [41] Spain	n = 18; m Healthy 38.2 ± 11.5 yrs BMI 24.7 ± 2.9 kg/m^2^ LDL 129 ± 44 mg/dL	2.7 vs. 366 mg/kg 25 mL/day Crossover 3 wks.	LDL-c change by phenol content: 2.7 mg/kg: 6.4 ± 2.8 mg/dL vs. 366 mg/kg: −6.3 ± 4.8 mg/dL; *p* = 0.028
Hernaez, A. J Nutr 2015 [53] Spain	N = 25; m Healthy 32.3 ± 11.2 yrs BMI-NA Weight = 78.2 ± 10.9 kg LDL-c 100.4 ± 38.6 mg/d	2.7 vs. 366 mg/kg 25 mL Crossover 3 wks.	LDL-c and composition change by phenol content: LDL-c: NS difference Total LDL particles: 2.7 mg/kg: 4.7 ± 22.0% vs. 366 mg/kg: −11.9 ± 12.0%; *p* = 0.013 Apo B 2.7 mg/kg +6.4 ± 16.6% vs. 366 mg/kg −5.9 ± 16.6%; *p* = 0.004
Martin-Pelaez, S. Eur J Nutr 2017 [62] Spain	N = 18; m Healthy 36 ± 11.1 yrs BMI 24.3 ± 3.2 kg/m^2^ LDL-c 125 ± 45 mg/dL	2.7 vs. 366 mg/kg 25 mL/day 65% oleuropein Crossover Double blind 3 wks.	LDL-c change by phenol content: 2.7 mg/kg 4.87 ± 4.13 mg/dL vs. 366 mg/kg −6.61 ± 4.12 mg/dL; *p* = 0.04
Khandozi, N. Int J Food Sci Nutr 2021 [54] Iran	N = 40 (m = 38) >1 CVD risk factor EVOO: 53.6 ± 7.6 yrs. Refined: 56.0 + 6.3 yrs. LDL-c: EVOO: 83.3 (69.4–97.2) Refined: 61.4 (55.8–73.2)	2–10 (refined) vs. 500–700 (EVOO) mg/kg 25 mL/day 6 wks.	LDL-c change: Refined: 4.3 (−1.2 to 9.9) vs. EVOO: −5.1 (−0.55 to −10.7); *p* = 0.011
Sarapis, K. Br J Nutr 2022 [68] Australia	N= 50; m = 34% Healthy 38.5 ± 13.9 yrs. BMI 24.7 ± 3.5 kg/m^2^ LDL-c: 115.8 ± 34.7 mg/dL	86 vs. 320 mg/kg 60 mL/day Crossover Double blind 3 wks	No between-group differences

^a^ m = male. ^b^ BMI = body mass index. ^c^ EVOO = extra virgin olive oil. ^d^ f = female. ^e^ BP = blood pressure. ^f^ HTN = hypertension. ^g^ BMI = body mass index. ^h^ VOO = virgin olive oil. ^i^ FU = follow-up. ^j^ NCI = National Cancer Institute. ^k^ LF = low-fat diet. ^l^ CHD = coronary heart disease. ^m^ CI = confidence interval. ^n^ CAD = coronary artery disease.

**Table 4:** The effect of extra virgin olive oil on high-density lipoprotein cholesterol (HDL-c) compared with diets with other fats, low-fat diets, and olive oil varying by phenol content.

First Author, Journal, Year, Country	Population	Intervention/Design	Outcomes
**Other fats**			
Madigan, C. Diabetes Care 2000 [59] Ireland	N = 11; m ^a^ Type 2 DM 56.0 ± 2.5 yrs A1c: 5.7 ± 0.86% BMI ^b^ 27.7 ± 2.6 kg/m^2^ HDL-c = 42.3 ± 11.6 mg/dL	EVOO ^c^ vs. sunflower oil (SO) 30 mL/d Crossover 2 wks.	HDL-c comparison by intervention: SO 42.5 ± 22.5 vs. EVOO 42.5 ± 11.6; NS
Perona, J.S. Clin Nutr 2004 [65] Spain	N = 62; m/f ^d^ 31 nl BP ^e^; 31 HTN 84 ± 7.4 yr BMI 28.8 ± 5.2 kg/m^2^ HDL-c: NA	VOO ^f^ (232 mg/kg) vs. sunflower (SO) 60 g/day Crossover 4 wks.	HDL-c comparison by intervention and by nl BP and HTN nl BP: VOO 48.1 ± 14.9 mg/dL vs. SO 57.6 ± 17.8 mg/dL; *p* < 0.01 HTN: VOO 51.2 ± 17.2 mg/dL vs. SO 54.0 ± 18.7; *p* > 0.01
Kontogianni, M.D. Metabolism 2013 [56] Greece	N = 37 (m = 8) Healthy 25.6 ± 5.9 years BMI 21.9 ± 2.5 kg/m^2^ HDL-c: EVOO: 60.2 ± 15.4 mg/dL Flaxseed: 61.8 ± 11.6 mg/dL	EVOO vs. Flaxseed oil 15 mL Crossover Single blind 6 wks.	HDL-c change from baseline: EVOO: 60.2 ± 15.4 mg/dL vs. FU 59.8 ± 397.7 * mg/dL Flaxseed 61.8 ± 11.6 mg/dL vs. FU 60.6 ± 11.6 mg/dL *p* = 0.69 * Value provided in Table 2 of article; possibly an error
Maki, K.C. J Clin Lipidol 2015 [60] USA	N = 54 m/f Healthy 53.8 ± 1.3 yrs. BMI: 28.2 ± 0.5 kg/m^2^ HDL 47.4 ± 1.7 mg/dL	EVOO or corn oil (CO) 4 tbs/day Crossover Double blind 21 days	Compared with baseline HDL: EVOO −1.7% CO −3.4% *p* = 0.192
Kruse, M. Mol Nutr Food Res 2015 [58] Germany	N = 19; m Healthy EVOO 58.0 ± 2.6 yrs BMI 29.2 ± 0.7 kg/m^2^ HDL-c: 43.7 ± 3.1 mg/dL Rapeseed/canola 52.0 ± 2.3 yrs BMI: 29.7 ± 0.87 kg/m^2^ HDL-c 44.9 ± 2.3 mg/dL	EVOO or rapeseed (canola) oil (CO) 50 g/day 4 wks.	HDL-c change from baseline: EVOO 2.3 ± 1.9 vs. −2.7 ± 1.5 mg/dL; *p* = 0.61
Khaw, K.T. BMJ Open 2018 [55] UK	N = 91; m/f d Healthy Coconut oil (n = 28) 59.1 ± 6.1 yrs BMI: 25.5 ± 4.5 kg/m^2^ HDL-c: 77.2 ± 19.3 mg/dL Butter (n = 33) 61.5 ± 5.8 yrs BMI: 24.8 ± 3.5 yrs HDL-c: 73.4 ± 19.3 mg/dL EVOO (n = 30) 59.1 ± 6.4 yrs BMI: 25.0 ± 4.5 yrs. HDL-c: 69.5 ± 19.3 mg/dL	Coconut oil vs. butter vs. EVOO 50 g/day 4 wks.	HDL-c change from baseline: Coconut oil: + 10.8 mg/dL vs. butter +3.5 + 10.4 mg/dL vs. EVOO 3.9 + 5.8 mg/dL; *p* = 0.009 Compared with EVOO: Coconut oil +6.2 mg/dL (CI ^g^ 95%1.2 to 10.8); *p* < 0.05
Galvao Candido, F. Eur J Nutr 2018 [51] Brazil	N = 41; f Control: 27.2 ± 6.1 yrs BMI: 29.7 ± 0.6 kg/m^2^ HDL-c: 21.4 ± 1.0 mg/dL EVOO: 26.8 ± 5.0 yrs BMI: 30.5 ± 0.6 kg/m^2^ HDL-c: 23.6 ± 1.3 mg/dL	EVOO vs. soybean oil (control) 25 mL/day Double-blind 9 wks.	Change in HDL-c (mean, SE): EVOO: −0.54 ± 0.54 mg/dL vs. control: −1.3 ± 0.54 mg/dL; *p* = 0.38
**Low-fat diet**			
Paniagua, J.A. J Am Coll Nutr 2007 [64] Spain	N = 11; f = 7 Offspring ob/type 2 DM A1c 6.0 ± 0.5 62 ± 9 yrs BMI 32.6 ± 7.8 kg/m^2^ HDL-c: NA	SAT: 38% fat, 23% SFA, 47% CHO MFA: 38% fat, 73 g/d EVOO 47% CHO LF ^h^: 65% CHO, 20% fat Food provided Crossover 4 wks.	HDL comparison of EVOO with LF: EVOO 47 ± 5.4 mg/dL vs. LF 42 ± 4.2 mg/dL; *p* < 0.05
Flynn, M.M. J Women’s Health 2010 [49] USA	N = 28; f Breast cancer survivors 59.2 ± 6.1 yrs BMI 27.9 ± 2.8 HDL-c- NA	EVOO 3 tbs./day vs. <30% fat (NCI diet) Crossover 8 wks.	HDL-c comparison by intervention: EVOO 68 ± 12 mg/dL vs. LF: 64 ± 13; *p* = 0.001
Sola, R.M. Atherosclerosis 2011 [69] Spain	PREDIMED N = 551; m/f Type 2 DM or ≥3 CHD risk factors EVOO (n = 181) 69.3 ± 6.2 yrs. BMI 29.8 ± 4.3 kg/m^2^ HDL-c 51.9 ± 12 mg/dL LF (n = 177) 69.7 ± 6.3 yrs. BMI 30.1 ± 4.3 kg/m^2^ HDL-c 54.4 ± 11.3 mg/dL	EVOO 4 tbs./day Nuts 30 gr/day LF 3 months	HDL comparison of EVOO with LF: EVOO vs. LF: +2.1 (95% CI 0.9 to 3.2) mg/dL; *p* = 0.001
Flynn, M.M. J Cancer Therapy 2017 [50] USA	N = 18; m Prostate cancer on surveillance 66.6 ± 5.9 yrs BMI 30.9 ± 2.7 kg/m^2^ HDL-c: 46.3 ± 10.9 mg/dL	EVOO (625 mg/kg total phenols) 3 tbs./day vs. Prostate Cancer Foundation diet (LF diet) Crossover 8 wks.	HDL-c comparison by intervention: EVOO: 45.6 ± 13.5 mg/dL vs. PCF 44.4 ± 13.9 mg/dL; *p* = 0.12
Campos, V.P. J Hum Nutr Diet 2020 [40] Brazil	N= 204; m/f Control = 67 (49% m) Nuts = 68 (55% m) Olive oil = 69 (51% m) Stable CAD Normal HDL NS between groups at Baseline	Control diet = 25% fat Nuts, pecans = 30 g/day Olive oil, total phenol = 172 mg/kg, 30 mL/day 12 weeks of study	Comparison between the groups: NS difference for change in HDL-c
**Refined olive oil**			
Kozic, D.S. Med Sci Monit 2015 [57] Croatia	N= 35; HIV+ men 18–75 ys. old BMI: 23.0–27.9 kg/m^2^ HDL-c (by diet order): 46.3 to 50.2 mg/dL	Refined or EVOO 50 mL/day Crossover 20 days	HDL: Refined 50.2 ± 0.0 EVOO 50.2 ± 0.0 *p* = 0.884
**Total phenol**			
Marrugat, J. Eur J Nutr 2004 [61] Spain	N = 30; m Healthy 54.8 ± 21.4 to 61.0 ± 19.2 yrs BMI: <25.0 kg/m^2^ HDL-c: 54.1 ± 11.6 to 57.9 ± 11.6 mg/dL	Refined vs. 68 vs. 150 mg/kg 25 mL/day Crossover Double blind 3 wks.	HDL by phenol content: Refined: 61.0 ± 13.1 vs. 62.5 ± 13.1 mg/dL; NS 68 mg/kg: 60.6 ± 13.1 vs. 60.2 ± 11.9 mg/dL; NS 150 mg/kg: 60.6 ± 11.2 vs. 63.7 ± 12.4 mg/dL; *p* = 0.029
Weinbrenner, T. J Nutr 2004 [33] Spain	N = 12; m Healthy 21.1 yrs; (20–22 yrs) BMI 22.9 ± 1.7 kg/m^2^ HDL-c: (by diet order) 44.8 ± 3.5 to 46.3 ± 3.1	10 vs. 133 vs. 486 mg/dL 25 mL/day Crossover Double blind 4 days	HDL comparison by phenol content: 10 mg/kg: 46.3 + 3.1 → 48.3 + 3.5 mg/dL 113 mg/kg: 44.7 + 3.5 → 47.9 + 3.9 mg/dL * 486 mg/kg: 46.3 + 3.1 → 49.4 + 3.1 mg/dL * * *p* < 0.05 Linear trend: *p* < 0.05
Covas, M.I. Ann Intern Med 2006 [42] Spain	N = 200; m Healthy BMI < 25.0 kg/m^2^ 23.7–24.0 mg/m^2^ HDL-c: (by diet order) 47.0 ± 11.0 to 47.9 ± 11.3 mg/dL	2.7 vs. 164 vs. 336 mg/kg 25 mL/day Crossover 3 wks.	HDL-c compared with baseline: 2.7 mg/kg = +0.9 mg/dL 164 mg/kg = +1.2 mg/dL 336 mg/kg = +1.7 mg/dL *p* = 0.018
Castaner, O. Am J Clin Nutr 2012 [41] Spain	N = 18; m Healthy 38.2 ± 11.5 yrs BMI 24.7 ± 2.9 kg/m^2^ HDL 47 ± 10 mg/dL	366 vs. 2.7 mg/kg 25 mL/day Crossover 3 wks.	HDL: NS difference
Martin-Pelaez, S. Eur J Nutr 2017 [62] Spain	N = 18; m Healthy 36 ± 11.1 yrs BMI 24.3 ± 3.2 kg/m^2^ HDL 46 ± 10 mg/dL	2.7 vs. 366 mg/kg 25 mL/day 65% oleuropein Crossover Double blind 3 wks.	HDL 2.7 mg/kg 2.59 ± 1.40 vs. 366 mg/dL 0.49 ± 1.40; *p* = 0.26
Khandozi, N. Int J Food Sci Nutr 2021 [54] Iran	N= 40; m = 38 >1 CVD risk factor EVOO: 53.6 + 7.6 yrs Refined: 56.0 + 6/3 yrs HDL-c: EVOO: 43.9 mg/dL (39.1 to 53.9) Refined: 37.2 mg/dL (33.6 to 40.7)	2–10 (refined) vs. 500–700 (EVOO) mg/kg 25 mL/day 6 wks.	HDL-c change: Refined: 1.60 mg/dL (−0.24 to 2.45) vs. EVOO: −1.47 mg/dL (−9.96 to 1.96); *p* = 0.11
Sarapis, K. Br J Nutr 2022 [68] Australia	N = 50; 34% m 39 ± 14 yrs HDL 57.9 ± 11.6 mg/dL	86 vs. 320 mg/kg 60 mL/day Crossover Double blind 3 wks.	No between-group differences.
**HDL2; HDL function**			
Hernaez, A. Arterio Thromb Vasc Biol 2014 [35] Spain	N = 47; m Healthy 33.5 ± 10.9 yrs HDL-c: 52 ± 11 mg/dL	2.7 vs. 366 mg/kg 25 mL/day Crossover 3 wks.	HDL comparisons by phenol content: HDL: NS difference total (values NA) Percent change from baseline: HDL-c efflux capacity: 2.7 mg/kg: −2.34 vs. 366 mg/kg +3.05; *p* = 0.042 HDL2: 366 mg/kg: +15% vs. baseline; *p* = 0.01 vs. 2.7 mg/kg; *p* = 0.05

^a^ m = male. ^b^ BMI = body mass index. ^c^ EVOO = extra virgin olive oil. ^d^ f = female. ^e^ BP = blood pressure. ^f^ VOO = virgin olive oil. ^g^ CI = confidence interval. ^h^ LF = low-fat diet.

**Table 5:** The effect of extra virgin olive oil on fasting blood glucose (FBG), insulin, and HOMA-IR compared with diets with other fats, low-fat diets, and olive oil varying by phenol content.

First Author, Journal, Year, Country	Population	Intervention/Design	Outcomes
**Other fats**			
Madigan, C. Diabetes Care 2000 [59] Ireland	N = 11; m ^a^ type 2 DM 56.0 ± 2.5 yrs BMI ^b^ 27.7 ± 2.6 kgm^2^ FBG ^c^ NA A1c 5.7 ± 0.8%	EVOO ^d^ vs. sunflower oil (SO) 30 mL/d Crossover 2 wks.	FBG comparison by intervention: SO: 153.0 ± 14.4 mg/dL vs. EVOO: 136.8 ± 12.6 mg/dL; *p* < 0.01 Insulin (mU/L): SO: 2.23 ± 0.48 mU/L vs. EVOO: 1.97 ± 0.38 mU/L; *p* < 0.001
Kontogianni, M.D. Metabolism 2013 [56] Greece	N = 37 (m = 8) Healthy 25.6 ± 5.9 years BMI 21.9 ± 2.5 kg/m^2^ FBG < 90 mg/dL: approximately 60 mg/dL	EVOO vs. Flaxseed oil 15 mL Crossover Single blind 6 wks.	FBG: comparison by intervention: EVOO: baseline 86.2 + 7.2 mg/dL vs. FU ^f^ 85.3 + 5.4 mg/dL Flaxseed: baseline 87.1 + 7.2 mg/dL vs. FU 86.6 + 7.2 mg/dL *p* = 0.50
Kruse, M. Mol Nutr Food Res 2015 [58] Germany	N = 19 m Healthy EVOO ^d^ 58.0 + 2.6 yrs BMI 29.2 ± 0.7 kg/m^2^ FBG 107.5 ± 6.4 mg/dL Rapeseed/canola oil 52.0 ± 2.3 yrs BMI 29.7 ± 0.9 kg/m^2^ FBG 103.5 ± 3.4 mg/dL	EVOO or rapeseed (canola) oil (CO) 50 g/day 4 wks.	Change from baseline by intervention: FBG: EVOO −15.8 ± 6.8 mg/dL vs. CO −4.8 ± 2.4 mg/dL; *p* = 0.153 Insulin: EVOO 0.3 ± 0.8 mU/L vs. CO −2.2 ± 0.8 mU/L; *p* = 0.058 HOMA-IR ^e^: EVOO 0.3 ± 0.5 vs. CO −0.5 ± 0.2; *p* = 0.154
Galvao Candido, F. Eur J Nutr 2018 [51] Brazil	N = 41; f ^g^ Normotensive EVOO: 26.8 ± 5.0 yrs BMI: 30.5 ± 0.60 kg/m^2^ Control: 27.2 ± 6.1 yrs. BMI:29.7 ± 0.6 kg/m^2^	EVOO vs. soybean (control) 25 mL/day Double-blind 9 wks.	Change by intervention (mean, SE) Glucose: EVOO: −0.11 + 0.39 vs. control: −0.13 ± 0.05; *p* = 0.81 Insulin: EVOO: −4.31 ± 5.9 vs. control: 3.82 ± 35.6; *p* = 0.06 HOMA-IR EVOO: −0.19 ± 0.22 vs. control: 0.08 ± 1.15: p0.054
Khaw, K.T. BMJ Open 2018 [55] UK	N= 91 m/f Healthy EVOO (n = 32) 59.1 ± 6.4 yr BMI: 25.0 ± 4.5 kg/m^2^ FBG: EVOO: 5.4 ± 0.5 mmol/L Coconut oil: 5.3 + 0.4 mmol/L Butter: 5.4 ± 0.5 mmol/L	EVOO vs. coconut oil vs. butter 50 g/day 4 wks.	Mean change from baseline by group: FBG: EVOO: −0.06 ± 0.49 vs. coconut oil: −0.05 ± 0.49 vs. butter: 0.02 ± 0.48; *p* = 0.68
**Low-fat diet**			
Estruch, R. Ann Intern Med 2006 [46] Spain	PREDIMED N = 722; m/f Type 2 DM or >3 CHD risk factors EVOO (n = 257) 68.6 ± 6.9 yrs BMI: 29.7 ± 4.1 kg/m^2^ FBG, insulin: NA LF (n = 257) 69.5 + 6.1 yrs BMI: 30.2 + 4.3 kg/m^2^ FBG, insulin: NA	EVOO vs. nuts vs. (control) LF EVOO = 4 tbs/day 3 months	Change in EVOO intervention vs. LF: FBG: −7.02 mg/dL (CI ^i^ −13.0 to −1.3); *p* = 0.017 Insulin: −2.4 mU/L (CI −3.9 to −0.06); *p* < 0.001 HOMA: −0.91 (CI −1.40 to −0.46); *p* < 0.001
Paniagua, J.A. J Am Coll Nutr 2007 [64] Spain	N = 11; f ^g^ = 7 Offspring ob/type 2 DM. A1c 6.0 ± 0.5% 62 ± 9 yrs BMI 32.6 ± 7.8 kg/m^2^ FBG: 98.5 ± 9.0 mg/dL Insulin: 12.6 ± 3.8 mU/L	EVOO: 38% fat, EVOO 73 g 47% CHO LF ^h^: 65% CHO, 20% fat SAT: 38% fat, 23% SFA, 47% CHO Food provided Crossover 4 wks.	Comparison by intervention: EVOO 90.4 ± 2.5 mg/dL* vs. LF 90.0 ± 2.3 mg/dL * vs. SAT 99.0 ± 18.0 mg/dL; *p* < 0.05 Insulin (mU/L): EVOO 8.7 ± 1.8 mU/L vs. LF 10.8 ± 1.8 mU/L vs. SAT 9.2 ± 1.4 mU/L; *p* = 0.30 HOMA-IR: EVOO 2.3 ± 0.3 * vs. LF 2.5 ± 0.4 vs. SAT 2.7 ± 0.4; *p* < 0.05 * EVOO vs. LF
Flynn, M.M. J Women’s Health 2010 [49] USA	N = 28; f Breast cancer survivors 59.2 ± 6.1 yrs BMI 27.9 ± 2.8 kg/m^2^ FBG: NA	EVOO vs. <30% fat (NCI ^j^ diet) 3 tbs./day EVOO Crossover 8 wks.	Comparison by intervention: FBG: EVOO 91.0 ± 7.7 mg/dL vs. NCI: 90.0 ± 7.0 mg/dL; *p* = 0.87 Insulin: EVOO 10.4 ± 3.8 vs. NCI 9.9 ± 3.4 uU/mL; *p* = 0.40
Domenech, M. Hypertension 2014 [43] Spain	PREDIMED N = 235 m/f Type 2 DM or ≥3 CHD risk factors EVOO (n = 78) 66.2 ± 5.8 yrs BMI: 29.5 ± 3.9 kg/m^2^ FBG: 123.1 mg/dL (95% CI 114.6 to 131.6) Nuts (n = 78) 67.2 ± 5.3 yrs BMI: 29.5 ± 3.9 kg/m^2^ FBG: 119.6 mg/dL (95% CI 111.8 to 127.4) LF (n = 75) 66.2 ± 6.2 yrs BMI 30.4 ± 3.5 kg/m^2^ FBG: 113.8 mg/dL (95% CI 106.2 to 121.5)	EVOO vs. nuts vs. LF EVOO = 50 mL/day 1 year	Change in FBG by intervention: EVOO: −6.13 mg/dL * (95% CI −11.62 to −0.64) vs. Nuts: −4.61 mg/dL (95% CI −9.82 to 0.60) vs. LF: 3.51 mg/dL (95% CI −0.51 to 7.54); *p*= 0.016 * Significantly different vs. LF
Flynn, M.M. J Cancer Therapy 2017 [50] USA	N = 18; m Prostate cancer on surveillance 66.6 ± 5.9 yrs BMI: 30.9 ± 2.7 kg/m^2^ FBG: NA	EVOO (625 mg/kg) vs. Prostate Cancer Foundation (PCF) diet 3 tbs./day EVOO Crossover 8 wks.	Comparison by intervention: FBG: EVOO 99.1 ± 9.6 vs. PCF 104.9 ± 9.9 mg/dL; *p* = 0.01 Insulin: EVOO 11.5 ± 4.4 mU/L vs. PCF 13.7 ± 7.0 mU/L; *p* = 0.02 HOMA-IR: EVOO 2.9 ± 1.2 vs. 3.6 ± 2.1; *p* = 0.02
Dos Santos, J.L. Eur J Clin Nutr 2022 [44] Brazil	N = 204 Control = 67 Nuts = 68 Olive oil = 69 40–60 yrs.	Control diet = 25% fat Nuts, pecans = 30 g/day Olive oil:172 mg/kg 30 mL/day 12 weeks of study	Comparison between groups for FBG, A1c, and fasting insulin: NS difference
**Refined olive oil**			
Kozic, D.S. Med Sci Monit 2015 [57] Croatia	N = 35; m HIV + 18–75 ys EVOO → refined Mean (CI 25–75%) BMI: 25.2 kg/m^2^ (23.3–27.9) FBG: 95.4 (91.8–90) Refined → EVOO Mean (CI 25–75%) BMI: 24.3 kg/m^2^ (23.0–26.0) FBG: 102.6 mg/dL (93.6–116)	EVOO vs. refined 50 mL/day Crossover 20 days	FBG comparison by intervention: EVOO 99 ± 1.8 mg/dL vs. refined 99 ± 1.8 mg/dL; *p* = 0.894
**Total phenol**			
Fito, M. Atherosclerosis 2005 [48] Spain	N= 40; m Stable CHD Refined → EVOO 69.8+ 8.4 yrs BMI: 28.0 + 3.0 kg/m^2^ FBG: 122.6 + 43.9 mg/dL EVOO → refined 66.0 + 8.9 yrs BMI: 27.0 + 3/1 kg/m^2^ FBG: 114.8 + 34.6 mg/dL	EVOO 161 mg/kg vs. 14.7 (refined) 50 mL/day Crossover 3 wks.	Comparison by phenol content: 161 mg/kg 119.7 ± 40.1 mg/dL vs. refined 116.3 ± 36.9 mg/dL; *p* = 0.171
Castaner, O. Am J Clin Nutr 2012 [41] Spain	N = 18; m Healthy 38.2 ± 11.5 yrs BMI 24.7 ± 2.9 kg/m^2^ FBG 87 ± 14 mg/dL	366 vs. 2.7 mg/kg 25 mL/day Crossover 3 wks.	FBG comparison by phenol content: 366 mg/kg 88 ± 11 mg/dL vs. 2.7 mg/kg 87 ± 11 mg/dL; *p* = 0.44
Martin-Pelaez, S. Eur J Nutr 2017 [62] Spain	N = 18; m Healthy 36 ± 11.1 yrs BMI 24.3 ± 3.2 kg/m^2^ FBG 88 ± 14 mg/dL	2.7 vs. 366 mg/kg 25 mL/day 65% oleuropein Crossover Double blind 3 wks.	FBG comparison by phenol content: 366 mg/kg: 1.00 ± 2.21 mg/dL vs. 2.7 mg/kg: 0.72 ± 2.21 mg/dL; *p* = 0.56

^a^ m = male. ^b^ BMI = body mass index. ^c^ FBG = fasting blood glucose. ^d^ EVOO = extra virgin olive oil. ^e^ HOMA-IR = Homeostatic Model Assessment of Insulin Resistance. ^f^ FU = follow-up. ^g^ f = female. ^h^ LF = low-fat diet. ^i^ CI = confidence interval. ^j^ NCI = National Cancer Institute.

**Table 6:** The effect of extra virgin olive oil on body weight compared with diets with other fats, low-fat diets, and olive oil varying by phenol content.

First Author, Journal, Year, Country	Population	Intervention/Design	Outcomes
**Other fats**			
Galvao, C.F. Eur J Nutr 2018 [51] Brazil	N = 41; f ^a^ Normotensive EVOO ^b^: 26.8 ± 5.0 yrs BMI ^c^ 30.5 ± 0.60 kg/m^2^ Control: 27.2 ± 6.1 yrs BMI 29.7 ± 0.6 kg/m^2^	EVOO ^d^ vs. soybean (control) 25 mL/day Double blind 9 wks.	Change by intervention: Body weight: EVOO −2.75 ± 0.38 kg vs. control −1.7 ± 0.47 kg; *p* = 0.09 Body fat (DXA ^e^): EVOO −2.4 ± 0.3 kg vs. control −1.3 ± 0.4 kg; *p* = 0.037
**Low-fat diet**			
Flynn, M.M. J Women’s Health 2010 [49] USA	N = 28; f Breast cancer survivors 59.2 ± 6.1 yrs BMI 27.9 ± 2.8 kg/m^2^	EVOO vs. <30% fat (NCI ^f^ diet) EVOO 3 tbs./day Crossover 8 wk. wt. loss 6-month FU	Percent of baseline weight lost by order of diets: EVOO first: −6.5 ± 1.6% vs. NCI first 4.6 ± 1.5%; *p* < 0.01
Flynn, M.M. J Cancer Therapy 2017 [50] USA	N = 18; m ^g^ Prostate cancer on surveillance 66.6 ± 5.9 yrs BMI 30.9 ± 2.7 kg/m^2^	EVOO 625 mg/kg vs. Prostate Cancer Foundation (PCF) diet EVOO 3 tbs./day Crossover 8 wk. wt. loss 6-month FU	Percent weight loss by diet: EVOO: −2.8 ± 3.7% vs. PCF −2.5 ± 3.1%; *p* = 0.86
Estruch, R. Ann Intern Med 2019 [45] Spain	PREDIMED N= 7447; m/f EVOO (n = 2543) 67.0 ± 6.2 yrs BMI 29.9 ± 3.7 kg/m^2^ LF (n = 2450) 67.3 ± 6.3 yrs BMI 30.2 ± 4.0 kg/m^2^	EVOO vs. nuts vs. (control) LF ^h^ EVOO = 50 mL/day 4.8 yrs.	EVOO compared with LF: Body weight (kg): −0.43; *p* = 0.044 Waist (cm): −0.55 cm; *p* = 0.048
**Total phenol**			
Castaner, O. Am J Clin Nutr 2012 [41] Spain	N = 18; m Healthy 38.2 ± 11.5 yrs BMI 24.7 ± 2.9 kg/m^2^	366 vs. 2.7 mg/kg 25 mL/day Crossover 3 wks.	BMI (kg/m^2^) comparison by phenol content: 2.7 mg/kg: 24.8 ± 2.8 kg/m^2^ (+0.13 ± 0.05) vs. 366 mg/kg: 24.7 ± 2.9 kg/m^2^ (−0.09 ± 0.08); *p* = 0.033
Martin-Pelaez, S. Eur J Nutr 2017 [62] Spain	N = 18; m Healthy 36 ± 11.1 yrs BMI 24.3 ± 3.2 kg/m^2^	2.7 vs. 366 mg/kg 25 mL/day 65% oleuropein Crossover Double blind 3 wks.	BMI changes by phenol content: 2.7 mg/kg: 0.11 ± 0.07 kg vs. 366 mg/kg: −0.06 ± 0.07 kg; *p* = 0.09

^a^ f = female. ^b^ EVOO = extra virgin olive oil. ^c^ BMI = body mass index. ^d^ EVOO = extra virgin olive oil. ^e^ DXA = Dual-Energy X-ray absorptiometry. ^f^ NCI = National Cancer Institute. ^g^ m = male. ^h^ LF = low-fat diet.
